# Antifungal prophylaxis and novel drugs in acute myeloid leukemia: the midostaurin and posaconazole dilemma

**DOI:** 10.1007/s00277-020-04107-1

**Published:** 2020-06-08

**Authors:** Jannik Stemler, Philipp Koehler, Christian Maurer, Carsten Müller, Oliver A. Cornely

**Affiliations:** 1 Department I of Internal Medicine, Faculty of Medicine and University Hospital Cologne, Center for Integrated Oncology Aachen Bonn Cologne Duesseldorf (CIO ABCD), Excellence Center for Medical Mycology (ECMM), University of Cologne, Cologne, Germany; 2 Cologne Excellence Cluster on Cellular Stress Responses in Aging-Associated Diseases (CECAD), University of Cologne, Cologne, Germany; 3 German Centre for Infection Research, Partner Site Bonn-Cologne, Cologne, Germany; 4 Centre of Pharmacology, Therapeutic Drug Monitoring, Faculty of Medicine, Cologne, University Hospital of Cologne, Cologne, Germany; 5 Clinical Trials Centre Cologne (ZKS Köln), University of Cologne, Cologne, Germany

**Keywords:** Targeted therapy, Therapeutic drug monitoring, Blood levels, Personalized medicine, Protein kinase inhibitor

## Abstract

With the advent of new targeted drugs in hematology and oncology patient prognosis is improved. Combination with antifungal prophylaxis challenges clinicians due to pharmacological profiles prone to drug–drug interactions (DDI). Midostaurin is a novel agent for *FLT3*-TKD/-ITD^mut^-acute myeloid leukemia (AML) and metabolized via cytochrome P450 3A4 (CYP3A4). Posaconazole is a standard of care antifungal agent used for prophylaxis during induction treatment of AML and a strong CYP3A4 inhibitor. Concomitant administration of both drugs leads to elevated midostaurin exposure. Both drugs improve overall survival at low numbers needed to treat. The impact of CYP3A4-related DDI remains to be determined. Severe adverse events have been observed; however, it remains unclear if they can be directly linked to DDI. The lack of prospective clinical studies assessing incidence of invasive fungal infections and clinical impact of DDI contributes to neglecting live-saving antifungal prophylaxis. Management strategies to combine both drugs have been proposed, but evidence on which approach to use is scarce. In this review, we discuss several approaches in the specific clinical setting of concomitant administration of midostaurin and posaconazole and give examples from everyday clinical practice. Therapeutic drug monitoring will become increasingly important to individualize and personalize antineoplastic concomitant and antifungal treatment in the context of DDI. Pharmaceutical companies addressing the issue in clinical trials may take a pioneer role in this field. Other recently developed and approved drugs for the treatment of AML likely inhere potential of DDI marking a foreseeable issue in future treatment of this life-threatening disease.

## Background

Acute myeloid leukemia (AML) is the most common acute leukemia type in adults [1, 2]. Until recently, treatment of AML relied on intensive chemotherapy regimens associated with high treatment-related mortality. Over the last decade, several new drugs have been developed representing a major change in the management of AML. Among them, especially inhibitors targeting a mutation of the fms-like tyrosine kinase 3 (*FLT3*), present in 20–30% of AML patients, are of interest [3–5]. The *FLT3-*mutation is associated with a shorter overall survival and decreased disease-free survival as compared to patients with wild type (*FLT3*^WT)^, thus being a major target to improve prognosis in this population [6].

AML patients can have disease-associated impaired neutrophil function, and intensive chemotherapy regularly induces prolonged neutropenia exposing them at highest risk for developing invasive fungal disease (IFD) [7, 8]. Several clinical trials have established antifungal prophylaxis with triazoles as standard of care in patients with AML, allogeneic stem cell transplantation (HSCT), and graft-versus-host disease (GvHD) [9–12]. Posaconazole is a second-generation triazole with activity against a broad-spectrum of yeasts and molds, and it is a strong cytochrome p450 (CYP) 3A4 inhibitor [13, 14].

Midostaurin, previously known as PKC412, is a first-generation tyrosine kinase inhibitor (TKI) for the treatment of *FLT3*-TKD/-ITD^mut^ AML. It is also approved for aggressive systemic mastocytosis (ASM), systemic mastocytosis with associated hematological neoplasm (SM-AHN), and mast cell leukemia (MCL). The introduction of midostaurin as a targeted drug represents a paradigm shift in treating patients with *FLT3*-mutated AML.

However, concerns have come up that drug–drug interactions (DDI) with posaconazole threaten the use of each of the two substances and data to support the decision is currently lacking. This review aims to summarize available evidence of potential DDI with these two drugs and to offer solutions for their concomitant use in clinical routine to maximize patient benefit.

## The case of midostaurin and posaconazole

### Midostaurin: development, dosage, and adverse events

Midostaurin was approved for treatment of *FLT3*-TKD/-ITD^mut^ AML in the USA in 2017 and in the European Union in 2018 after the RATIFY trial proved that the addition of midostaurin to standard induction treatment significantly increased overall and event-free survival of patients with AML [15].

According to the prescribing information, midostaurin should be administered from day 8 to 21 of each 21-day induction (7 + 3 cytarabine and anthracycline) and consolidation chemotherapy cycle in a dosage of 50 mg twice daily. In patients with complete remission (CR), continuous daily midostaurin intake is recommended for 1 year or until relapse and in case of allogeneic HSCT the drug is to be stopped 48 h prior to conditioning chemotherapy [16]. Midostaurin is a substrate to CYP3A4, which converts the drug into further active metabolites, CPG6221 and CGP52421. All three substances were shown to inhere multi-kinase inhibition activity especially in clonal heterogenous AML [17, 18]. The metabolites provide additional antileukemic activity by targeting peripheral blasts non-selectively [19]. The described effects apparently add up for midostaurin effectiveness.

Midostaurin demonstrated a low toxicity profile in early clinical trials in solid tumor entities [20]. The relatively safe profile paved the way for evaluation among other populations, including AML. Adverse events (AEs) are mostly low-grade gastrointestinal toxicities such as nausea, vomiting, and diarrhea, but also QTc prolongation and interstitial lung disease can occur (Table 1) [16, 21]. Early clinical trials also observed two fatal pulmonary AEs of unclear etiology despite thorough workup [22]. In the extension of the trial, two more cases of severe pulmonary edema occurred. Affected patients had excessive midostaurin levels while being administered azole compounds. Thereafter, enrollment of patients receiving azoles was suspended and a clear chest X-ray was made an inclusion criterion. Subsequently, no other pulmonary AEs were observed [23]. Strong interpatient variability of plasma concentrations was noted [22].

**Table 1:** Selected adverse events of midostaurin and management [16]

Adverse event	Proposed management
Nausea/vomiting	Administration with food Administration with antiemetic
QTc prolongation	Electrocardiogram monitoring Maintain potassium and magnesium within normal limits
Interstitial lung disease and pneumonitis Pleural effusion Pulmonary hemorrhage	Monitor closely for pulmonary symptoms Discontinue midostaurin in patients who develop symptoms of interstitial lung disease

*QTc* corrected QTc interval

A retrospective sub-analysis of the RATIFY trial showed no significant increase in midostaurin- or midostaurin-metabolite-related AEs in patients receiving strong CYP3A4 inhibitors. These were defined as fluconazole, ciprofloxacin, voriconazole, and posaconazole with the latter being administered in less than 40% of patients, even during induction treatment. This exposure-safety-matched control analysis revealed earlier onset of first clinically noted adverse events (CNAE) in patients with higher midostaurin and CPG6221 exposure [24]. Of note, a 1.44-fold increase in midostaurin exposure was observed in patients who had strong CYP3A4 inhibitors administered concomitantly compared to those who had not. CYP3A4 inducing medications, such as rifampin, showed even a 10-fold decrease of midostaurin levels, leading to the clear recommendation to avoid concomitant administration of such agents [25]. Interestingly, administered daily doses of midostaurin varied broadly between early clinical trials with a maximum dose up to 225 mg [23, 26].

Current Food and Drug Administration and European Commission package inserts on midostaurin underline the risk of pulmonary toxicity and recommend withdrawal of the drug upon observation of severe pulmonary events [27, 28].

### Posaconazole-based antifungal prophylaxis

Azole-based prophylaxis has proven effective in prevention of IFD in long-term neutropenic patients, allogeneic HSCT, or patients with GvHD and is strongly recommended by numerous international guidelines [29–32]. In particular posaconazole was successfully investigated in AML patients during induction remission chemotherapy and reduced incidence of IFD from 8 to 2%, now being a worldwide life-saving standard in this population [9]. Administration of posaconazole begins during the first days of administration of chemotherapy and ends upon recovery of neutropenia in a standard dosage of once daily 300 mg tablets [33]. It has an enhanced spectrum of activity, also covering *Fusarium* spp. and Zygomycetes [34, 35]. Triazoles are inhibitors of the cytochrome p450 enzyme, especially CYP3A4, with posaconazole belonging to the group of strong inhibitors [14]. The drug is available as oral tablet, oral suspension, and intravenous (i.v.) formulation of which the tablet and i.v. are proven to be safe and effective in risk populations [11, 12].

Especially the tablet formulation of the drug inheres this effect being of more reliable pharmacokinetics than the oral suspension formulation. Its distinctly higher peroral bioavailability is associated with higher serum concentrations [36].

Several factors influencing posaconazole exposure in patients with AML/MDS have been identified, among them concomitant use of proton-pump inhibitors, diarrhea or otherwise altered gastrointestinal function, high weight, and co-administration of chemotherapy [37, 38]. Therapeutic drug monitoring (TDM) is advised by current guidelines to optimize exposure and clinical efficacy and for cases of clinical failure [29, 30]. Other antifungals are frequently used for prophylaxis in different patient populations, some of them despite being proved inferior compared to posaconazole (Table 2) [33, 39–42].

**Table 2:** Selected antifungals, CYP3A4 impact, and clinical considerations [33, 39–42]

Antifungal agent	CYP3A4 impact	Clinical considerations for antifungal prophylaxis
Posaconazole [33]	Strong inhibition	QTc prolongation Oral solution associated with low absorption and plasma level variation, TDM recommended Hepatic toxicity
Isavuconazole [39]	Moderate inhibition	QTc shortening Hepatic toxicity higher rate of breakthrough fungal infections when used for prophylaxis [80, 81]
Voriconazole [40]	Strong inhibition	QTc prolongation Vision changes Hepatic toxicity Hallucinations Long-term use associated with skin cancer
Micafungin [41]	Minor substrate	Well tolerated Only available intravenously Limited efficacy against molds
Caspofungin [42]	Minor substrate	Well tolerated Only available intravenously Limited efficacy against molds

*CYP3A4* cytochrome p450 3A4 enzyme, *QTc* corrected QT interval, *TDM* therapeutic drug monitoring, *i.v.* intravenous

### Drug–drug interactions and therapeutic drug monitoring

Over the years, numerous orally available anti-neoplastic drugs have become available. However, the medical team is confronted with new challenges while treating patients with these agents, in particular with potential DDI linked to the high number of CYP3A4-metabolized drugs [43, 44]. A retrospective study found potential DDI in 46% (total *n* = 900) of patients treated with oral anti-neoplastic therapy, of which 16% were classified as severe defined as requiring further interventions or being of harmful nature [45]. The high amount of suspected DDI underlines the clinical impact. It should alert clinicians not to indulge the promising results of novel anti-tumor therapies but also consider their side effects. DDI have constantly played a role in medical therapy also affecting hematology patients in the context of immunosuppressive agents, anti-infective drugs, and proton-pump inhibitors [46, 47].

In the case of midostaurin and posaconazole, both drugs are indispensable in the specific clinical setting. Each has been shown to improve overall survival, and numbers needed to treat (NNT) to save a life are low at fifteen and fourteen, respectively [9, 15, 48]. Inhibition of CYP3A4 increases midostaurin in vivo exposure. In pharmacological studies on healthy subjects receiving ketoconazole with concomitant midostaurin, a tenfold increase in area under the curve (AUC) and doubled plasma concentration (*C*_max_) were observed [25]. With midostaurin being the drug targeting the principal underlying disease and posaconazole considered part of supportive care, co-administration of triazoles with midostaurin could be discouraged. On the other side, it has been suggested to withhold midostaurin only during induction chemotherapy when risk for IFI is the highest [49]. However, this strategy deprives patients of the survival benefit of midostaurin as assessed in the RATIFY trial. Currently, non-evidence-based practice has become routine in this specific case and is displayed in Table 3. Triazoles have been a clear-cut standard for antifungal prophylaxis in hematology, but recommendations and subsequently clinical practice seem to drift apart due to fear of DDI. Standardized approaches are missing, and the lack of clear guidance in this specific setting has been pointed out previously [49]. This leads to a change in behavior of clinicians and leaves the decision of choice of antifungal agent to the treating team.

**Table 3:** Strategies for clinical use of antifungal prophylaxis in AML patients treated with midostaurin

Scenario/strategy	Pro	Contra	Recommendation by the authors
1. Administration of recommended dosage of midostaurin as of package insert and standard dosage antifungal prophylaxis with posaconazole. Monitor patient closely for AE(s).	- Antileukemic activity of midostaurin as assessed in clinical trials is assured	- Close monitoring of AEs (e.g., frequent ECG controls, clinical evaluation of pulmonary function) must be warranted [16] - Increased risk of midostaurin-related AE(s) is given	Moderately recommended This approach detects potential toxicity-related AE late
2. Dose reduction of midostaurin to ~ 50% during induction treatment while posaconazole is administered.	- Risk of early onset of AEs and generally AEs is most likely omitted	- Antileukemic activity of midostaurin is not warranted as assessed in clinical trials - Midostaurin dosage increase must be guaranteed when posaconazole is stopped - Non-adherence to azole prophylaxis or altered pharmacokinetics lead to low midostaurin exposure	Marginally recommended This approach potentially restricts the therapeutic effect of midostaurin while not providing efficacy monitoring
3. Switch antifungal prophylaxis to EC or other triazoles (Fluconazole, Itraconazole, Isavuconazole).	- EC do not exhibit a significant CYP3A4 inhibition - Isavuconazole shortens the QTc interval [78] - Isavuconazole: safe and effective [76]	- Limited power studies/transferred evidence of efficacy and safety for other antifungals from other patient populations available [64, 66, 68, 83] - Fluconazole/Itraconazole proved to be inferior in antifungal prophylaxis [9] - EC: administration only via i.v. route/minor penetration to central nervous system [71–74] - Isavuconazole: not available for low resource settings/cost	Marginally recommended Other antifungal agents than posaconazole have been proven inferior or provide similar CYP3A4 effects
4. Continue with recommended dosage of midostaurin and posaconazole as of package insert and measure drug levels via TDM of both drugs regularly.	- Determination of plasma/serum levels allows monitoring of prophylactic effectiveness of posaconazole and antileukemic activity of midostaurin [84] - Dose adaption according to measured level of midostaurin allows individualized dosage	- TDM method for determination of metabolites (CGP6221 and CGP52421) levels not yet available	Strongly recommended TDM allows close therapy monitoring and individualized dosing in the future. This strategy reflects the “Cologne approach”.

*AE* adverse event, *EC* echinocandins, *ECG* electrocardiogram, *TDM* therapeutic drug monitoring

To assess DDI, a rating and classification system has been developed [50]. Midostaurin-posaconazole DDI were stratified in the third of five categories defined as “monitor therapy” with clinical impact being unclear, but not requiring major therapy alteration [16, 44]. Additionally, several tools have been proposed including multidisciplinary interaction checks, computerized order entry systems, software-based guidance, clinical monitoring, and therapeutic drug monitoring (TDM) [50]. TDM seems to be the most exact and efficient tool in order to assess optimal dosing of drugs with a narrow therapeutic window and DDI. TDM of posaconazole and other antifungals is already established and clinically indicated [51]. In this setting, it should aim to determine a sufficient exposure of the drug. Subsequently, TDM of midostaurin is flattering to adapt and personalize dose depending on the individual inhibitory CYP effect by the antifungal. The large interpatient variability in a drug with a probably narrow therapeutic window like midostaurin supports the use of TDM [52].

Individualization of dosage by means of TDM has been highly recommended in transplant patients on immunosuppressants with antifungals [50]. TDM contributes to patient safety and optimal management with targeted agents in hematology and might be a new option for administration of midostaurin and posaconazole. The lack of a prospective sub-study, which specifically assesses interactions of antifungal prophylaxis and midostaurin by comparing drug levels, questions the reliability of retrospective data [24]. Since the described CNAEs were not clearly defined, and potential AEs as pulmonary edema and QTc-prolongation can be life-threatening or even fatal, this topic requires urgent further evaluation. Given that future approval procedures for novel agents, especially in oncology, might require interaction studies, any study assessing potential DDI in detail as primary objective constitutes to a pioneer position. Additionally, the understanding of pharmacological mechanisms underlying the DDI of midostaurin and posaconazole has to be increased and shall be subject to further pharmacological investigations.

## Strategies for antifungal prophylaxis in AML patients treated with midostaurin

Multidisciplinary approaches are essential in current management of hematology patients at risk for potential DDI. Recommendations are given for some novel hematologic drugs despite midostaurin. For example, the use of combination treatment of venetoclax with hypomethylating agents becomes more frequent since it showed promising results in patients with newly diagnosed or relapsed/refractory AML not eligible for intensive chemotherapy [53]. This treatment results in prolonged cytopenia exposing patient to IFD and therefore indicates the use of antifungal prophylaxis in this population [54]. With venetoclax being a CYP3A4 metabolized drug, a dose reduction of 75% is recommended while concomitant posaconazole prophylaxis is administered [55]. An AUC increase up to 8.8-fold was observed, and consistency with CYP3A4-mediated inhibition of venetoclax metabolism was concluded. Of note, this recommendation is based on a prospective sub-study, which included only twelve patients [56]. Nonetheless, this kind of DDI study marks a pioneer role in clinical trials aiming to license novel targeted treatments—especially in hematology. The retrieved evidence paved the way for modified clinical practice considerations in the specific setting, but is lacking for midostaurin [57]. Hence, strategies on how to handle patients on concomitant midostaurin and posaconazole are subject of paper arguments and expert opinion while clear guidance is lacking [49].

Several approaches have been proposed to best prevent potential DDI in concomitant administration of midostaurin and antifungals.

### Continue as recommended by manufacturer and closely monitor adverse effects

Administration of posaconazole concomitant to 50 mg twice daily of midostaurin represents the current approved standard for both drugs. These dosages have been investigated under trial conditions and should provide the necessary antileukemic activity [16, 33]. Midostaurin administration at approved dose requires close monitoring on AEs, especially cardiac and pulmonary, but no strict measures are advised by the manufacturer at the moment [16]. Upon occurrence of severe AEs, withdrawal of the drug is recommended. This approach comprises the increased probability to detect midostaurin-related severe AEs too late and expose patients to unpredictable risks. Several publications have recommended this approach assuming the risk of DDI not to be significantly increased [44, 58]. Other authors have raised awareness for the issue considering the risk of DDI clinically important. Thus, this approach remains to be investigated in a robust clinical trial and tested for validity.

### Dose reduction of midostaurin during induction remission treatment and concomitant posaconazole administration

Another approach consists in decreasing the dose of midostaurin to 50% (i.e., 25 mg b.i.d) during induction treatment, a strategy which has been proposed and implemented by leading hematologists [59]. On the one hand, this seems reasonable in reducing risk of dose excess of midostaurin and consecutive adverse effects and posaconazole remains as prophylactic agent. However, without local availability of midostaurin blood level monitoring or response in *FLT3-*expression, this strategy seems questionable considering the decreased antileukemic activity of potentially underdosed midostaurin. Monitoring *FLT3* expression has been evaluated but underlies polymerase chain reaction bias and requires bone marrow samples not being feasible on a very regular basis [60, 61]. Furthermore, non-adherence to prophylactic regimen or pharmacokinetic alterations can lead to decreased posaconazole blood levels and subsequently to lower midostaurin levels. Additionally, posaconazole is usually administered only during induction treatment while midostaurin is continued during consolidation until allogeneic HSCT or 1 year of maintenance or even further [16]. Therefore, once posaconazole prophylaxis is stopped, dose increase of midostaurin must be guaranteed in order to avoid underdosing and depriving AML patients of effective therapy.

### Switch antifungal prophylaxis to other antifungal agents

A third option in management is a change of class in antifungal prophylaxis. Hematology centers may decide to use echinocandins (EC) in patients receiving midostaurin. Micafungin is broadly used in children, in patients not eligible for posaconazole due to intolerability, and in patients undergoing allogeneic stem cell transplantation for *Candida*-directed prophylaxis [62, 63]. Micafungin prophylaxis has shown similar results compared to fluconazole in efficacy studies in similar patient populations [64, 65]. It has even been suggested as alternative prophylactic agent in AML [64, 66]. However, this recommendation is derived from studies with limited statistical power. Caspofungin has also been used as prophylactic agent in AML patients with mixed results [67, 68]. EC are the drugs of choice for invasive candidiasis; caspofungin is also approved for salvage treatment of invasive aspergillosis [42]. Nevertheless, they are commonly known to provide poor CNS penetration [69, 70]. Additionally, they lack of coverage against some *Mucorales* and *Fusarium* species, which are discovered with increasing frequency in patients with hematological malignancies [71–74]. Ultimately, they are only available intravenously.

Another appealing choice for primary antifungal prophylaxis is isavuconazole with only moderate CYP3A4 inhibition. It has been proven safe and effective at a 200 mg or 400 mg daily dose in AML and MDS patients [75, 76]. Isavuconazole is an extended spectrum triazole agent approved for treatment of invasive aspergillosis and mucormycosis [39, 77]. Pharmacological features seem to be favorable with very reliable absorption and 98% oral bioavailability. The additional effect of QTc-interval shortening makes this drug a serious alternative for concomitant administration with novel hematological therapies [78, 79]. However, the efficacy data for primary prophylaxis use are contradictory, especially because of a higher rate of breakthrough fungal infections than compared to voriconazole or posaconazole [80–82]. This observation seems to be the case especially for breakthrough invasive pulmonary aspergillosis [82]. Voriconazole seems to be a negligible option in this context since it is not considered superior to posaconazole as prophylactic agent and has similar CYP3A4 inhibitory effects [83].

### Continue as recommended by manufacturer and implement therapeutic drug monitoring

Finally, the fourth approach foresees implementing TDM for both midostaurin and posaconazole in patients receiving these drugs concomitantly and dose adaption according to measured drug levels.

In a first step, standard-of-care utilization of TDM needs to be implemented for patients receiving midostaurin. Highly sensitive methods for TDM of the drug are available, including UPLC-MS/MS (ultra performance liquid chromatography–tandem mass spectrometry) for simultaneous determination of midostaurin in serum and plasma matrix, but not broadly implemented in TDM units of hematology centers [84–86]. To date, reference ranges for novel drugs remain to be determined. In fact, this currently impedes the second step—a standardized dose adaption according to measured midostaurin level. However, obtained results may lead the way to optimized dosing. On the other side, TDM of midostaurin for individual dose adaptation is questionable, if metabolites cannot be measured at the same time. Given the potential anti-leukemic activity, a CYP3A4-mediated inhibition of the metabolization of midostaurin would also decrease the amount of metabolites and therefore their anti-leukemic effect [87].

This fourth strategy displays the “Cologne approach.” At our hospital, patients on midostaurin treatment undergo twice weekly TDM for both drugs simultaneously with the second step of dose adaption being under consideration. In the future, the availability and standardized use of TDM methods in the respective patient population may allow individualized dosing of oncological and anti-infective drugs. Overall, this last approach contributes to a personalized management of AML patients receiving midostaurin and posaconazole concomitantly and may provide the safest and most efficient way to avoid adverse events and outcomes.

Further approaches and additional measures to each strategy are conceivable. A pharmacodynamic assay to measure *FLT3*-inhibiting effects in plasma in vivo has been proposed. This approach can complement pharmacokinetic data by focusing on the target of a drug and not on a drug itself as conventional pharmacokinetic methods like TDM do [87]. Some hematology centers may consider avoiding triazole prophylaxis completely and instead use diagnostic-driven counteractions including galactomannan monitoring and chest CT in the initial workup for febrile neutropenia. At least during induction remission chemotherapy, triazole antifungal prophylaxis seems to be of higher importance than *FLT3-*inhibitor administration. A survival benefit has been shown for posaconazole during this highest-risk period to develop IFD, whereas midostaurin seems to not play a role in CR attainment during initial treatment and might be more beneficial on a long-term perspective [88]. Reduction of antifungal dose cannot be recommended generally since their efficacy has been linked strongly to sufficient plasma concentrations [89].

## Future developments

Antifungal prophylaxis becomes more complex in modern AML therapy as with any hematological malignancy. On the one hand, this is due to emerging fungal pathogens with resistance to antifungal agents used for prophylaxis [90, 91] and breakthrough fungal infections [92]. On the other hand, novel drugs for AML treatment comprising increased interaction potential with triazoles will be more frequently administered. Antifungal prophylaxis might be individualized according to the given anti-leukemic agent [44].

The use of CYP3A4 metabolized drugs will expand in the future. The clinical efficacy and safety of midostaurin in *FLT3*^WT^ AML are currently investigated in an ongoing Phase III trial (NCT03512197) as well as the prolonged treatment and application in other populations [59, 93, 94]. New-generation *FLT3* inhibitors are already approved or currently in trial, such as gilteritinib, quizartinib, or lestaurtinib [95–97]. Further multikinase inhibitors might become available with expanded approval for second- or third-line treatment, relapsed/refractory AML, and other indications. Recently, other drugs, which underlie a CYP3A4-mediated metabolism, have been approved, like the isocitrate dehydrogenase (IDH) inhibitors ivosidenib and enasidenib or the sonic hedgehog inhibitor glasdegib [98–101] and more agents are under development [102]. Unfortunately, potential DDI are frequently not assessed in clinical trials. Enrolled patient populations do not necessarily represent a real-world population complicating routine use upon approval [103]. Expected approval of these drugs and expanded use of midostaurin will increase clinical use and has to raise awareness in the treating medical team as potential DDI other than with antifungals while on concomitant midostaurin treatment have been identified [104]. In the specific case of midostaurin and posaconazole, it remains important to emphasize that clinicians should be reassured that posaconazole prophylaxis does neither represent a contraindication to administer midostaurin nor the other way around.

A personalized-medicine approach includes standard of care therapeutic drug monitoring for antifungal agents and antileukemic drugs simultaneously and following dose adaptions according to drug levels [52]. TDM already plays a role in oncology in drugs with a narrow therapeutic window to increase efficacy and reduce toxicity. However, determination of the optimal management and routine use of TDM for dose adaptation remains to be investigated in real-life clinical trials. TDM methods have not been established broadly at hematology centers. This is also of high importance in orally available drugs and other drugs undergoing CYP3A4 metabolization. CYP3A4 interactions are a subject to clinical practice in nearly all medical specialties and being investigated broadly. Future monitoring of CYP3A4 could be standardized by routinely determining 4-ß-hydroxycholesterol (4βHC), a marker measuring the activity of this CYP enzyme. It has been proposed for that purpose mostly for patients treated with strong CYP-inducers, but also inhibitors [105, 106]. Another recent discovery of CYP3A4 inhibitors preventing *FLT3-*TKI resistance by inhibiting CYP-expressing bone marrow stromal cells in vivo seems of interest to rather promote CYP3A4 inhibitors in patients on *FLT3* inhibitors in the future [19]. Novel antifungal agents are upcoming and might also represent future options for antifungal prophylaxis. However, until this point optimal management of patients receiving drugs which inheres potential of DDI must be warranted.

Novel targeted drugs improve patient prognosis in hematology and oncology. Combination with antifungal prophylaxis challenges clinicians due to pharmacological profiles favoring drug–drug interactions. Severe AEs have been observed; however, in current evidence it remains unclear if they can be directly linked to DDI. Especially *FLT3* inhibitors are promising in the present and future treatment of AML. It appears timely to evaluate their clinical impact on DDI and their outcome. Several management strategies have been proposed, but evidence on which approach to use is scarce and the lack of robust clinical studies assessing incidence of IFD and clinical impact of DDI pave the way to neglecting a life-saving standard. Pharmaceutical companies addressing this issue in phase III and IV trials may take a pioneer role in this field. TDM will become increasingly important to individualize and personalize combination antineoplastic and antifungal treatment.
